# Methylenetetrahydrofolate Reductase Gene *C677T* Polymorphism–Dietary Pattern Interaction on Hyperhomocysteinemia in a Chinese Population: A Cross-Sectional Study

**DOI:** 10.3389/fcvm.2021.638322

**Published:** 2021-06-24

**Authors:** Song Leng, Ai Zhao, Jian Zhang, Wei Wu, Qian Wang, Shan Wu, Li Chen, Qiang Zeng

**Affiliations:** ^1^Health Management Institute, The Second Medical Center & National Clinical Research Center for Geriatric Diseases, Chinese People's Liberation Army General Hospital, Beijing, China; ^2^Health Management Center, The Second Hospital of Dalian Medical University, Dalian, China; ^3^Vanke School of Public Health, Tsinghua University, Beijing, China; ^4^Department of Nutrition and Food Hygiene, School of Public Health, Peking University, Beijing, China

**Keywords:** hyperhomocysteinemia, methylenetetrahydrofolate reductase, *MTHFR C677T*, dietary pattern, interaction

## Abstract

**Background and aim:** Hyperhomocysteinemia (Hhcy) has been recognized as a risk factor of several chronic diseases. There is accumulating evidence that both genetic and dietary factors had a notable impact on the risk of Hhcy. The present study aims to investigate the interaction effect on Hhcy between methylenetetrahydrofolate reductase (MTHFR) gene *C677T* polymorphism and dietary intake.

**Methods:** Data were collected in a cross-sectional survey conducted in China; 3,966 participants with complete information on sociodemographic characteristics, anthropometric measurements, and dietary intake were included in the analyses. Dietary patterns were identified by factor analysis combined with cluster analysis. Blood samples were collected and *MTHFR C677T* genotypes were tested. Both the multiplicative statistical model and the additive model were conducted to investigate the interactive effects.

**Results:** Proportions of *MTHFR C677T* genotypes among participants were 29.2% for *TT*, 47.4% for *CT*, and 23.4% for *CC*. Three dietary patterns were identified, namely, the balanced pattern, the snack pattern, and the high-meat pattern. Compared with the balanced pattern, the other two patterns were associated with an elevated risk of Hhcy [the snack pattern: odds ratio (OR) 1.2, 95% confidence interval (CI) 1.0–1.5; the high-meat pattern: OR 1.3, 95% CI 1.1–1.6] after adjustment for age group, gender, residential region, and *MTHFR C677T* genotypes. A multiplicative interaction between the high-meat pattern and *MTHFR 677TT* genotype was observed, and synergistic effects between both the snack pattern and the high-meat pattern with *MTHFR 677TT* were identified.

**Conclusion:** Our results indicated that *MTHFR C677T* polymorphism and dietary patterns had interactive effects on Hhcy among the Chinese population. Subsequent targeted and appropriate dietary guidelines should be recommended for high-risk populations or patients of Hhcy carrying specific genotypes.

## Introduction

Hyperhomocysteinemia (Hhcy) has been identified as a risk factor of stroke, heart attack (1), atherosclerosis (2), Alzheimer's disease, age-related macular degeneration, and some disastrous cancers (3–5). According to a meta-analysis published in 2015, which included 60,754 Chinese subjects aged 3-97 years, the pooled prevalence of Hhcy was 27.5% (6). It is noteworthy that Hhcy prevalence appeared to be rising in the recent 5 years, which was reported between 34.4 and 67.7% across China from 2015 to 2019 (7–11).

Homocysteine (Hcy) is an amino acid generated metabolically by the S-adenosylmethionine-dependent transmethylation pathway (12). Transsulfuration, an alternative and essential route for Hcy catabolism, is initiated by the action of cystathionine β-synthase (CBS), a B6-dependent enzyme. 5,10-Methylenetetrahydrofolate (5,10-MTHF) reductase (MTHFR), a key enzyme during folate/B_12_-dependent remethylation pathway, converts 5,10-MTHF to 5-MTHF. For humans, a reduction in the activity of MTHFR owing to the single nucleotide *677C*→*T* variant has been associated with an elevated serum Hcy level and a higher risk of Hhcy (13, 14). As one meta-analysis targeted at a healthy population in South Asia showed, the serum Hcy level of those carrying the *MTHFR 677TT* genotype was significantly higher than that of *CC* genotype carriers (15). The odds ratio of Hhcy was also found higher for participants with the *CT* or *TT* genotypes, than the *CC* genotype in a cross-sectional study among the Chinese population (8). Besides, there is also accumulating evidence that dietary intake has a notable impact on the metabolic balance of Hcy, which could be mediated by affecting the levels of substrates, coenzymes, or products (16). Among Chinese general adults, two cross-sectional studies revealed negative associations between the consumption of fruits or vegetables and the odds of Hhcy (9, 17), while another study suggested that a higher frequency of eating fish was correlated with a lower serum Hcy level (18). Although both genetic and dietary factors affected the metabolism pathways of Hcy, there were no population-based studies exploring the interaction effects between them so far. Clarifying possible interaction effects undoubtedly plays an important role in the dietary intervention to high-risk populations or patients of Hhcy.

Therefore, our study aims to investigate the interaction effects on Hhcy between MTHFR gene *C677T* polymorphism and dietary intake.

## Materials and Methods

### Study Design and Participants

This study was based on a cross-sectional survey conducted from November 2014 to July 2015 in a general hospital in Beijing, China. Persons who came to the Department of Health Management in the surveyed hospital for an annual physical examination were invited to take part in an additional questionnaire survey that collected information on general characteristics, dietary intake, and lifestyle behaviors. The inclusion criteria adopted in the current study were being at the age of 20 years old or above, not using folic acid supplements or drugs that affect folic acid or vitamin B metabolism, and having stopped using these supplements or drugs for over 6 months. The exclusion criteria were being unconscious or unable to recall the survey contents; being pregnant or in the lactation period; and having serious liver, kidney, or other severe or inherited metabolic diseases (e.g., phenylketonuria). A total of 4,172 participants joined in this survey. The data were accessed on March 20, 2020. After excluding individuals who did not finish the survey (*n* = 121), who had missing values on key variables (*n* = 76), and whose *MTHFR C677T* genotypes failed to be determined (*n* = 9), 3,966 participants from 33 provinces of China were included in our analyses.

### Assessment of Dietary Intake and Dietary Pattern Analysis

A semiquantitative Food Frequency Questionnaire (FFQ) was used to collect information on participants' dietary intake during the past 12 months. Amounts or frequencies of food consumption were aggregated into 10 food groups, namely, cereals, meat and poultry, vegetables and fruits, fish and other aquatic products, milk and dairy products, eggs, legumes, desserts and candies, fried foods, and pickles and smoked foods. Daily cereal intake was expressed as grams per day ( ≤ 100, 100-200, 200-500, >500 g/day), and consumption of other foods was expressed as days per week (<1, 1-2, 3-4, 5-7 days/week).

Dietary patterns were derived using factor analysis combined with cluster analysis, a two-step approach that has been previously used (19). Firstly, factor analysis was conducted to identify major dietary factors. Since dietary intake data were ordinal, a polychoric correlation matrix was created in the factor analysis using the package psych (20). A combination of scree plot and eigenvalue >1 was used to determine the number of factors to extract. We kept the first three factors with a minimal eigenvalue of 1.23. Factor scores were calculated, and factor loadings were shown in the supporting information (Supplementary Table 1). The first factor showed high loadings on fried foods, desserts and candies, and pickles and smoked foods and was labeled as the snack factor. The second factor showed high loadings on meat and poultry and was labeled as the animal-meat factor. The third factor showed high loadings on eggs, legumes, milk and dairy products, and vegetables and fruits and was labeled as high-protein factors. Subsequently, factor scores were used in cluster analysis. A cluster tree was drawn to determine the number of clusters to identify. It turned out that the three-clusters option was optimal according to the cluster tree. Then, we set the number of clusters as three in the K-means cluster analysis, and all participants were grouped into three dietary patterns. The first pattern was characterized by positive scores on all factors and was named the balanced pattern. The second pattern showed a positive score on the snack factor and negative scores on the animal-meat factor and was named the snack pattern. The third pattern showed positive scores on the animal-meat factor and negative scores on other factors and was labeled as the high-meat pattern.

### Measurement of Hhcy

Blood samples were collected in the morning after fasting for over 8 h. All samples were centrifuged within 2 h, and serums were stored at −80°C. Plasma Hcy was analyzed by HPLC (LC-9A, Shimadzu Corp., Kyoto, Japan) with fluorescence detection (F-1080, Hitachi Ltd., Tokyo, Japan). Serum Hcy ≥15 μmol/L was diagnosed as Hhcy (21).

### Determination of *MTHFR C677T* Polymorphism

QIAamp DNA Mini Kit (Cat. No. 51304, Germany) was used to extract DNA from the whole blood. Gene chip hybrid analysis determined the of *MTHFR C677T* polymorphism. All the procedures obeyed the introductions of the BaiO genotype detecting gene array kit and equipment (BaiO Technology Corp., Shanghai, China).

### Assessment of Covariates

Covariates including gender, age, marital status, residential region, and work-related physical activity were obtained by the questionnaire. Anthropometric measurements, including waist, weight, and height, were conducted by nurses in the morning, and body mass index (BMI) was computed as weight (kg)/[height (m)]^2^.

### Statistics

The characteristics of the participants were presented as percentages, with differences across groups compared using chi-square tests for categorical variables. For continuous variables, means and SDs and Student's *t*-tests were used. Logistic regression models were conducted to estimate the association between dietary patterns and Hhcy, with adjustment for age group (20–40, 40–50, 50–60, or 60–75 years), gender (male or female), residential region (southern or northern China), and genotypes (*MTHFR 677CT/CC* or *TT*).

Subgroup analyses were conducted separately in participants carrying different *MTHFR C677T* genotypes. Sensitivity analyses were conducted by additionally adjusting for waist and BMI. In addition, we used both the multiplicative statistical model and the additive model to investigate interactive effects between dietary patterns and *MTHFR C677T* polymorphism on the incidence of Hhcy. In the multiplicative model, an interaction term between the dietary patterns and *MTHFR C677T* genotypes was added to the logistic model, and the coefficient of the interaction term represented the magnitude of the multiplicative interactive effect. In the additive model, a new nominal variable used in the logistic regression was generated according to combinations of the dietary patterns and *MTHFR C677T* genotypes, taking the first dietary pattern and *CT/CC* genotype as the reference group. Relative excess risk due to interaction (RERI) and attributable proportion due to interaction (AP) were estimated by published function (22).

As both *MTHFR C677T* genotypes and dietary patterns were nominal variables, to make it easier to interpret and understand the interactive analysis results, we recorded *MTHFR C677T* genotypes into a binary variable (*CT/CC* into 0 and *TT* into 1) and conducted stratified analysis according to the dietary patterns. We first included the balanced pattern and the snack pattern in one regression model, followed by the balanced pattern and the high-meat pattern in another one.

All the statistics were conducted in R 3.6.2. Statistical significance was defined as *P* < 0.05.

## Results

### *MTHFR C677T* Polymorphism and Its Association With General Characteristics

Of all the 3,966 participants, 25.3% were diagnosed with Hhcy. The percentages of participants carrying different *MTHFR C677T* genotypes were 29.2% for *TT*, 47.4% for *CT*, and 23.4% for *CC*. Participants diagnosed with Hhcy were more likely to carry the *MTHFR 677TT* genotype, be male, live in northern China, and have a higher waist and BMI (Table 1).

**Table 1:** Differences in the general characteristics between participants with and without hyperhomocysteinemia.

**Variable**	**Hcy <15**	**Hcy ≥ 15**	***P***
Number of participants	2,962	1,004	
*MTHFR C677T* genotype			
*TT*	20.9	53.8	<0.001
*CT*	52.2	33.5	
*CC*	26.9	12.7	
Gender			
Female	37.1	10.8	<0.001
Male	62.9	89.2	
Age (years)			
20–40	13.1	10.0	0.055
40–50	43.9	45.3	
50–60	36.1	36.7	
60–75	6.9	8.0	
Marital status			
Married	96.7	97.0	0.619
Unmarried, divorced, widow, and others	3.3	3.0	
Residential region			
Southern China	11.9	6.0	<0.001
Northern China	88.1	94.0	
Work-related physical activity^a^			
Sedentary work	56.6	57.7	0.517
Light or hard physical work	43.4	42.3	
Waist (cm)	88.2 ± 10.7	91.6 ± 9.4	<0.001
Body mass index (kg/m^2^)	25.3 ± 3.4	26.0 ± 3.2	<0.001

*Categorical variables were presented as percentage, and continuous variables were shown as mean ± SD. For categorical variables, differences between groups were tested by chi-square, and for continuous variables, Student's t-test was used*.

*MTHFR, methylenetetrahydrofolate reductase*.

a *Three missing values*.

### Dietary Pattern and Its Association With Hhcy

Of all the participants, 41.8% followed the balanced pattern, 23.9% followed the snack pattern, and 34.3% followed the high-meat pattern. Compared with the balanced pattern, both the snack pattern and the high-meat pattern were associated with an elevated risk of Hhcy after adjustment for covariates. In the subgroup analysis by *MTHFR C677T* genotypes, among individuals with the *TT* genotype, the associations were consistent with the combined population; however, in those with the *CT* or *CC* genotype, the associations did not reach the significant threshold (Table 2). In the sensitivity analyses, the associations between dietary patterns and Hhcy did not change after additionally adjusting for waist and BMI (Supplementary Table 2).

**Table 2:** Association between dietary patterns and hyperhomocysteinemia.

**Genotype**	**The balanced pattern**	**The snack pattern**		**The high-meat pattern**	
		**OR (95% CI)**	***P***	**OR (95% CI)**	***P***
Combined (*n* = 3,966)					
Crude	Ref	1.0 (0.8, 1.2)	0.841	1.3 (1.1, 1.5)	0.005
Adjusted^a^	Ref	1.2 (1.0, 1.5)	0.046	1.3 (1.1, 1.6)	0.005
*MTHFR 677TT* (*n* = 1,159)					
Crude	Ref	1.0 (0.7, 1.3)	0.987	1.8 (1.3, 2.3)	<0.001
Adjusted^a^	Ref	1.5 (1.1, 2.2)	0.011	1.7 (1.3, 2.3)	<0.001
*MTHFR 677CT/CC* (*n* = 2,807)					
Crude	Ref	0.9 (0.7, 1.2)	0.448	1.1 (0.9, 1.4)	0.445
Adjusted^a^	Ref	1.0 (0.8, 1.4)	0.752	1.1 (0.9, 1.4)	0.514

*Logistic regression models were conducted to investigate the association between dietary patterns and hyperhomocysteinemia*.

*OR, odds ratio; CI confidence interval; Ref, reference; MTHFR, methylenetetrahydrofolate reductase*.

a *Age group (20–40, 40–50, 50–60, or 60–75 years), gender (male or female), residential region (southern or northern China), and genotypes (MTHFR 677CT/CC or TT; only in the combined model) were adjusted*.

### Interaction Between Dietary Patterns and *MTHFR C677T* Polymorphism

**Figure 1:**
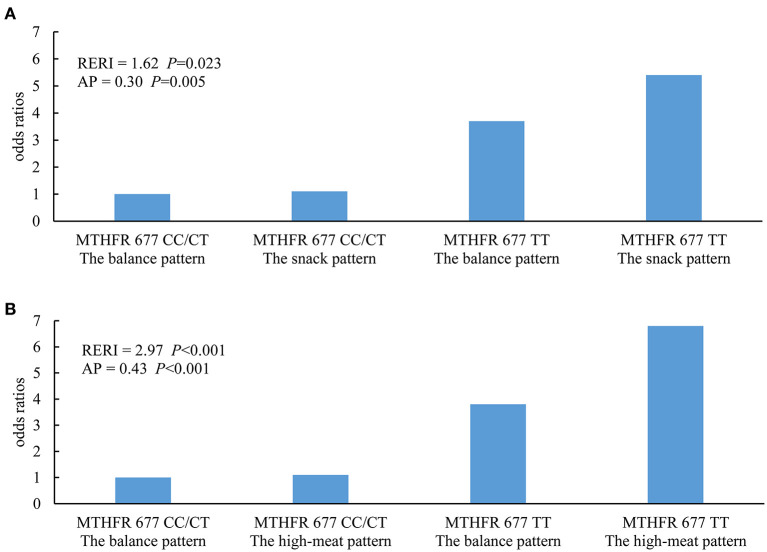
Additive interaction between dietary patterns and *MTHFR C677T* genotypes. **(A)** Results of the balanced pattern vs. the snack pattern were presented. **(B)** Results of the balanced pattern vs. the high-meat pattern were presented. RERI, relative excess risk due to interaction; AP, attributable proportion due to interaction. Additive models were conducted to estimate the interactive effects, in which age group (20–40, 40–50, 50–60, or 60–75 years), gender (male or female), and residential region (southern or northern China) were adjusted.

In the multiplicative models, an interaction between the *MTHFR 677TT* genotype and the high-meat pattern was observed (Table 3). In the additive models, synergistic effects of both the snack pattern and the high-meat pattern with the *MTHFR 677TT* genotype were observed (Figure 1).

**Table 3:** Interaction between dietary patterns and *MTHFR C677T* genotypes in relation to the incidence of hyperhomocysteinemia in the multiplicative model.

**Variables**	**The snack pattern**		**The high-meat pattern**	
	**OR (95% CI)**	***P***	**OR (95% CI)**	***P***
Genotype				
*MTHFR 677CC/CT*	Ref		Ref	
*MTHFR 677TT*	3.7 (2.9, 4.8)	<0.001	3.8 (3, 4.9)	<0.001
Dietary pattern				
The balanced pattern	Ref		Ref	
The snack pattern/the high-meat pattern	1.1 (0.8, 1.4)	0.601	1.1 (0.8, 1.3)	0.671
Interaction term	1.4 (0.9, 2.1)	0.158	1.7 (1.2, 2.5)	0.005

*Logistic regression models were conducted to investigate the association between dietary patterns and hyperhomocysteinemia. Covariates, including age group (20–40, 40–50, 50–60, or 60–75 years), gender (male or female), and residential region (southern or northern China), were adjusted*.

*OR, odds ratio; CI confidence interval; Ref, reference; MTHFR, methylenetetrahydrofolate reductase*.

## Discussion

Male, *MTHFR 677TT* genotype, larger waist, higher BMI, and residing in northern China were found to be associated with a higher risk of Hhcy. As for dietary factors, persons adhering to the snack pattern or the high-meat pattern were more likely to suffer Hhcy, compared with the balanced pattern. To our knowledge, this study was the first population-based cross-sectional survey to report interaction effects between genetic factors and dietary patterns on Hhcy in China. Both additive interaction effects and multiplicative effects were observed; however, differences in interactions captured between the two approaches should be noted.

The susceptibility to the Hhcy of *MTHFR 677TT* genotype carriers has been extensively documented (8, 13, 15). Likewise, our study found that the *TT* genotype frequency in Hhcy patients was higher than in non-patients. The *TT* genotype frequency in our study, 29.2%, was in the range of other researches in China, but higher than in other countries (8, 23–26). One study showed geographical gradients of the *TT* genotype frequency also existed across China (27), although our study did not further explore. The *TT* genotype frequency was significantly higher among northern populations and ranged from the lowest values (6.4%) in Hainan (southern) to the highest values (40.8%) in Shandong (northern) (27). Accordingly, geographical variations of *TT* genotype served as one important reason for regional differences in the Hhcy prevalence among countries and across China.

Apart from genetic factors, the serum Hcy level was closely dependent on nutritional factors. Numerous studies have indicated that Hhcy was related to dietary intake (16, 17, 28, 29). As there was no dietary resource of Hcy, it is generally considered that dietary intake indirectly exerted an influence on serum Hcy *via* its metabolism pathways. On one hand, serum levels of folate and vitamin B_12_ could be altered by the intake of food rich in them, such as fresh fruits and vegetables (30). Both folate and vitamin B_12_ were involved in the remethylation of Hcy to methionine. Two cross-sectional studies among Chinese general adults revealed a negative associations between consumption of fruits or vegetables and the odds of Hhcy (9, 17). A double-blinded, randomized clinical trial performed on an Italian population with mild Hhcy suggested that, compared with baseline and placebo, the serum Hcy level significantly decreased after daily additional 150 μg food folate mainly from increased intake of fruit and vegetables for 13 weeks (16). Another 5-month intervention study from Norway demonstrated that a reduction in the serum Hcy level was inversely related to an increment in folate intake due to increased consumption of whole-grain bread (28). On the other hand, dietary intake might affect the serum Hcy level by other mechanisms. The frequency of fish consumption less than three times per week was found to be correlated with a higher serum Hcy level in the Chinese population (18). Another study showed that the serum Hcy level of vegetarians was evidently higher than that of omnivores (31). The possible mechanism was that among vegetarians, dietary deprivation of methionine caused downregulation of the transsulfuration pathway at the CBS level in order to maintain the synthesis and functioning of many methionine-dependent metabolic processes. This adaptive response produced increased Hcy in biological fluids and, thus, enhanced methylation of Hcy to methionine. Antioxidation of dietary nutrients, such as vitamin C, vitamin E, and β-carotene, might also partly explain a lower serum Hcy level, which was observed in a cross-sectional survey of US adults (29). Most of the current studies focused on single food but ignored the integral effects of various dietary intake on Hcy *in vivo*. Therefore, from the perspective of dietary patterns, participants in our study were classified into three clusters to explore associations between Hhcy and dietary factors. Of the three dietary patterns, the balanced pattern seems to be healthier, as individuals following this pattern consumed a higher variety of foods. In contrast, the snack pattern and the high-meat pattern seem to be less healthy for the unbalanced dietary intake and the high consumption of some food groups, such as fried foods, desserts and candies, and so on. Among the whole population, the snack pattern and the high-meat pattern were associated with elevated risks of Hhcy after adjustment for confounders. The positive association of increased Hhcy risk with the snack pattern and the high-meat pattern might result from the high consumption of saturated fat and hydrogenated oil accompanying this relatively unhealthy diet (32, 33). Besides, we inferred that fresh vegetables and fruits, which are rich in folate and vitamin B, were the main reasons for the association of an elevated Hhcy risk with these two patterns.

In the further stratified analysis by *MTHFR C677T* genotypes, we observed differences in the associations of the risk of Hhcy with dietary patterns between the *TT* genotype and non-*TT* genotype subgroups. These differences implied that genetic polymorphism involved in folate metabolism modified the effect of dietary patterns on the serum Hcy level. In the *TT* genotype subgroup, the high-meat pattern was positively associated with increased risk of Hhcy, which was consistent with the combined analysis, while associations were not observed in individuals in the *CC/TT* subgroup. This could be explained by the interaction between the *MTHFR 677TT* genotype and the high-meat pattern, which was supported by both the multiplicative model and the additive model. The presence of the *MTHFR 677TT* genotype amplified the effect of the high-meat pattern on the risk of Hhcy so that the joint effect of these two factors significantly exceeded the sum of both. Polymorphisms of *MTHFR C677T* congenitally affect the serum Hcy level by determining the ability to produce biologically functional folate. Compared with *CC/TC* genotypes, the population with the *TT* genotype who have a lower level of 5-MTHF is more sensitive to factors that act on metabolism pathways of 5,10-MTHF, such as the high-meat pattern in this study.

The snack pattern was positively associated with the increased risk of Hhcy only in the *TT* genotype subgroup. However, there is a lack of interaction effects between the *MTHFR TT* genotype and the snack pattern in the multiplicative model, which indicated that the difference in sensitivity to the snack pattern across *MTHFR C677T* genotypes might be only explained by the additive interaction in the additive model after adjustment for potential confounders. This necessitates simultaneous consideration of additive and multiplicative interaction effects. In terms of statistics, the former represents the multiplication of independent effects, while the latter reflects the joint effects of different combinations of factors (34). Considering the complicated nature of biological mechanisms underlying interaction effects, these two kinds of statistical identification methods are able to complement each other (35, 36).

Several limitations in this study needed to be acknowledged. First, the cross-sectional design of this study did not allow inference of causality, so the associations drawn should be considered with caution. Although our study included subjects from 33 provinces in China, the uneven distribution across age groups and the single survey site selected limited generalizability; second, dietary intake was assessed by questions regarding the average intake of several food items, was quantified approximately in terms of grams per day or times per week, and was insufficient for further evaluation of participants' nutrient intakes and validation. Third, patients with chronic diseases, including hypertension, diabetes, dyslipidemia, and hyperuricemia, are apt to change their dietary habits. These dietary modifications might influence associations between Hhcy and dietary factors to some extent.

## Conclusions

Individuals with the *MTHFR 677TT* genotype are most susceptible to Hhcy. Given that Chinese had a higher proportion of *TT* genotype than other populations as well as the geographical difference in the variations of *MTHFR C677T* genotypes, detection of *MTHFR C677T* is beneficial to the prevention and treatment of Hhcy. However, the cost efficiency of a *MTHFR C677T* genotype screening among the Chinese population still needs to be evaluated. Subsequent targeted and appropriate dietary guidelines should be recommended for populations with specific genotypes, although more studies were still needed to elucidate interaction effects between dietary and genetic factors.

## Data Availability Statement

The datasets presented in this study can be found in online repositories. The names of the repository/repositories and accession number(s) can be found below: https://github.com/QZeng126/data.

## Ethics Statement

The studies involving human participants were reviewed and approved by Medical Ethics Committee of the Chinese People's Liberation Army General Hospital. The ethics committee waived the requirement of written informed consent for participation.

## Author Contributions

SL and QZ: conceptualization. SL, AZ, and WW: methodology. SL, JZ, and QW: writing of the original draft. SW, LC, and QZ: review and editing. All the authors have read and agreed to the published version of the manuscript.

## Conflict of Interest

The authors declare that the research was conducted in the absence of any commercial or financial relationships that could be construed as a potential conflict of interest.

## Abbreviations

Hhcy: hyperhomocysteinemia
Hcy: homocysteine
5-MTHF: 5-methyltetrahydrofolate
CBS: cystathionine b-synthase
MTHFR: methylenetetrahydrofolate reductase
FFQ: Food Frequency Questionnaire
BMI: body mass index
RERI: relative excess risk due to interaction
AP: attributable proportion due to interaction.
