# Supplementary material for: Methylenetetrahydrofolate Reductase Gene C677T Polymorphism–Dietary Pattern Interaction on Hyperhomocysteinemia in a Chinese Population: A Cross-Sectional Study
Source: Front Cardiovasc Med. 2021 Jun 24;8:638322. doi: 10.3389/fcvm.2021.638322 (PMC8263928; doi:10.3389/fcvm.2021.638322)
Supplement: Supplementary file 1 [file Table_1.DOCX]

Supplementary Table 1. Factor loadings

| Food groups | The snack factor | The animal-meat factor | The high-protein factor |
| --- | --- | --- | --- |
| Cereals | -0.01 | 0.19 | 0.01 |
| Meat and poultry | 0 | **1.00** | 0 |
| Vegetables and fruits | -0.33 | 0.35 | **0.41** |
| Fish and other aquatic products | 0.22 | 0.27 | 0.17 |
| Milk and milk products | 0.18 | 0.01 | **0.45** |
| Eggs | -0.01 | 0.24 | **0.69** |
| legumes | 0.12 | 0.10 | **0.57** |
| Desserts and candies | **0.56** | -0.06 | 0.13 |
| Fried foods | **0.89** | 0.02 | -0.02 |
| Pickles and smoked foods | **0.54** | 0.06 | -0.05 |
| %variance | 16.0 | 13.0 | 12.3 |

Absolute factor loadings greater than 0.4 were shown in bold.
