# Supplementary material for: Methylenetetrahydrofolate Reductase Gene C677T Polymorphism–Dietary Pattern Interaction on Hyperhomocysteinemia in a Chinese Population: A Cross-Sectional Study
Source: Front Cardiovasc Med. 2021 Jun 24;8:638322. doi: 10.3389/fcvm.2021.638322 (PMC8263928; doi:10.3389/fcvm.2021.638322)
Supplement: Supplementary file 2 [file Table_2.DOCX]

Supplementary Table 2. Sensitivity analysis of association between dietary patterns and hyperhomocysteinemia.

| Genotype | The balanced pattern | The snack pattern | |  | The high-meat pattern | |
| --- | --- | --- | --- | --- | --- | --- |
|  |  | OR (95%CI) | P |  | OR (95%CI) | P |
| Combined (n=3966) | Ref | 1.2(1.0,1.5) | 0.045 |  | 1.3(1.1,1.6) | 0.005 |
| *MTHFR 677TT* (n=1159) | Ref | 1.5(1.1,2.2) | 0.011 |  | 1.7(1.3,2.3) | <0.001 |
| *MTHFR 677CT/CC* (n=2807) | Ref | 1.0(0.8,1.4) | 0.741 |  | 1.1(0.9,1.4) | 0.504 |

OR, odds ratio; CI confidence interval; Ref, reference; MTHFR, methylenetetrahydrofolate reductase. Logistic regression models were conducted to investigate the association between dietary patterns and hyperhomocysteinemia. Covariates, including age group (20-40, 40-50, 50-60, or 60-75 years), gender (male or female), residential region (South or North of China), genotypes (*MTHFR 677CT/CC* or *TT*; only in the combined model), waist (cm), and body mass index (kg/m^2^), were adjusted.
